# The Effect of an Energy Window with an Ellipsoid Phantom on the Differential Defect Contrast on Myocardial SPECT Images

**DOI:** 10.3390/bioengineering9080341

**Published:** 2022-07-26

**Authors:** Ammar A. Oglat, Mohannad Adel Sayah

**Affiliations:** 1 Department of Medical Imaging, Faculty of Applied Medical Sciences, The Hashemite University, Zarqa 13133, Jordan; 2 Department of Radiography, Princess Aisha Bint Al-Hussein College of Nursing & Health Sciences, Al-Hussein Bin Talal University, P.O. Box 20, Ma’an 71111, Jordan; 0903006@ahu.edu.jo

**Keywords:** eccentricity phantom, defect contrast, energy window, myocardial study, SPECT scan

## Abstract

Good quality single-photon emission computed tomography (SPECT) images are required to achieve a perfect diagnosis and determine the severity of defects within the myocardial wall. There are many techniques that can support the diagnosis of defect formations in acquired images and contribute to avoiding errors before image construction. The main aim of this study was to determine the effect of energy width (15%, 20%, and 25%) on defect contrast in myocardial SPECT images correlated with the decentralization of positioning of a phantom. A phantom of polyethylene plastic was used to mimic the myocardial wall of the left ventricle. The phantom consists of two chambers, inner and outer. Two rectangular pieces of plastic were placed in anterior and inferior locations in the mid-region of the myocardial phantom to simulate myocardial infarction (defects). The average defect contrast for all phantom positions using 15% to 20% energy was (1.2, 1.6) for the anterior region and (1.1, 2) for the inferior region, respectively. Additionally, the energy window width was >25% with a large displacement of the positioning off center, leading to loss of the defect contrast in myocardial SPECT images, particularly in the inferior region. The study showed decreasing defect contrast in both locations, anterior and inferior, with increasing energy window width correlated with eccentricity positioning of the phantom on an imaging table.

## 1. Introduction

SPECT has been widely used in cardiac nuclear medicine imaging for nearly four decades. This non-invasive modality technique allows the examination of patients with known or suspected coronary artery abnormalities [1,2,3]. Over time, the use of this technique for the acquisition of cardiac images has become well established. The American Society of Nuclear Cardiology (ASNC) has published several guidelines for cardiac SPECT imaging [4,5,6]. Nowadays, better imaging properties have been achieved by technetium-99m (Tc-99^m^)-labeled radiotracers and new gamma cameras without any increase in scanning time [7,8,9].

SPECT can be utilized to define a patient’s pathologic status when cardiac symptoms cannot be explained by structural findings [10,11]. This disconnect between radiologic and clinical findings is frequently seen. SPECT has been routinely used to detect myocardial infarction in myocardial perfusion imaging [12].

Gamma cameras are used in SPECT imaging to obtain images from different angles from a patient on an imaging table injected with isotopes [13,14]. For SPECT images to be of perfect quality, the camera heads must avoid non-uniformity of isotope distribution within organs [15]. Variations in energy and uniformity of tomography reconstruction require more calibration for the center of rotation [16,17,18]. In the acquired SPECT image, limited energy causes scattering, and the scattering must be removed before correction [19,20]. Center of rotation error is one of the common reasons for artifacts that cause the non-uniformity of SPECT images [21]. The patient’s position on the imaging table should align the infected organ below the rotating gamma camera heads [22]. Many studies [23,24,25] in nuclear SPECT imaging focused on proper patient body positioning on an imaging table with gamma camera rotation.

SPECT with Tc-99^m^ uses parameters to obtain optimized imaging. Performance can be enhanced for visualizing perfusion distribution through a careful selection of parameters such as energy windows and positioning on an imaging table. This study seeks to test the different energy windows of Tc-99m on the defect contrast of myocardial SPECT images correlated with the positioning on an imaging table [23,24].

## 2. Methods

### 2.1. Phantom Design and Configuration

Fabricated ellipsoid phantoms of polyethylene plastic (*Z_eff_* = 7.5) were used for this study. The Zeff of polyethylene can be calculated using Equation (1) as follows:

(1) $$Z_{eff}=\left(a_{1\;}Z_{1\;}^{3}+a_{2\;}Z_{2}^{3}+\ldots \right)1/3\;$$

where

α is the fractional content of electrons of the *i*th element and

*Z* is the atomic number of the *i*th element.

On the basis of chemical composition (carbon and hydrogen) and Equation (1), the effective atomic number for polyethylene (C612_2_ H11_4_) is calculated as follows:

$$Z_{eff}={\;}^{3}\;\sqrt{2\times 6^{3}+4}\;Z_{eff}=7.5$$

Moreover, the number of electrons per gram ($N_{0}$) on a compound polyethylene can be calculated using Equation (2) as follows:

(2) $$N_{0}\frac{N_{A}\cdot \sum Z\;}{\sum A_{W\;}}\;$$

$$N_{0}=\frac{N_{A}\cdot \sum Z\left(C,H\right)}{\sum A_{W\;}\left(C,H\right)}\;N_{0}=\frac{6.023\times 10^{23}\left[2\times 12+4\times 1\right]}{\left[2\times 12+4\times 1\right]}\;N_{0}=3.44\times 10^{23}\;\mathrm{electrons}/g.$$

where

$N_{A}$ is Avogadro’s number

$A_{W\;}$ is the atomic weight and

*Z* is the atomic number.

Notably, the $\;N_{0}\;$ for water is 3.343 $\times 10^{23}\;$ electrons/g (Attix, 2008), whereas that for the cardiac muscle is 3.312 $\times 10^{23}\;.$ Considering the effective atomic number (*Z_eff_*) and electron densities (*N*_0_) of polyethylene, water, and cardiac muscle, it is concluded that polyethylene is a suitable material to simulate LV myocardial wall tissue.

**Figure 1:**
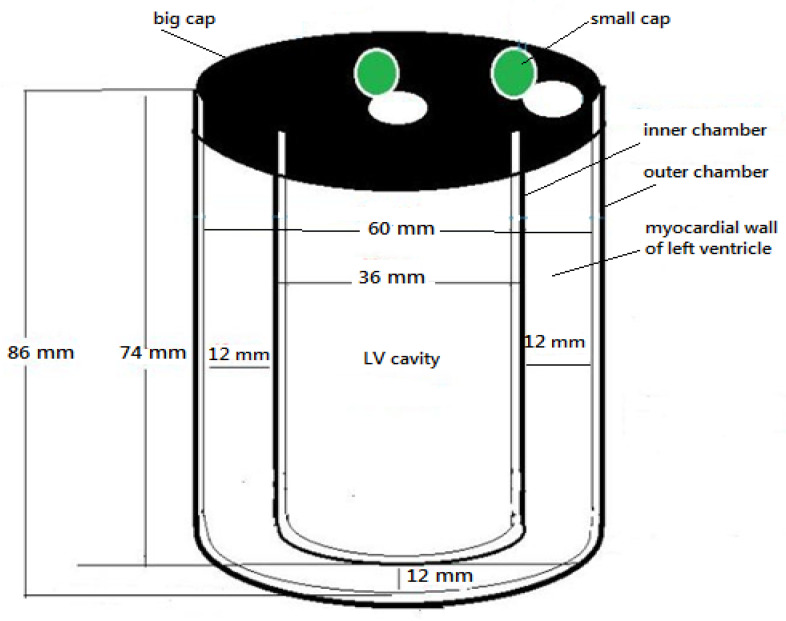
Schematic phantom to simulate myocardial wall of the left ventricle with two solid plastics.

**Figure 2:**
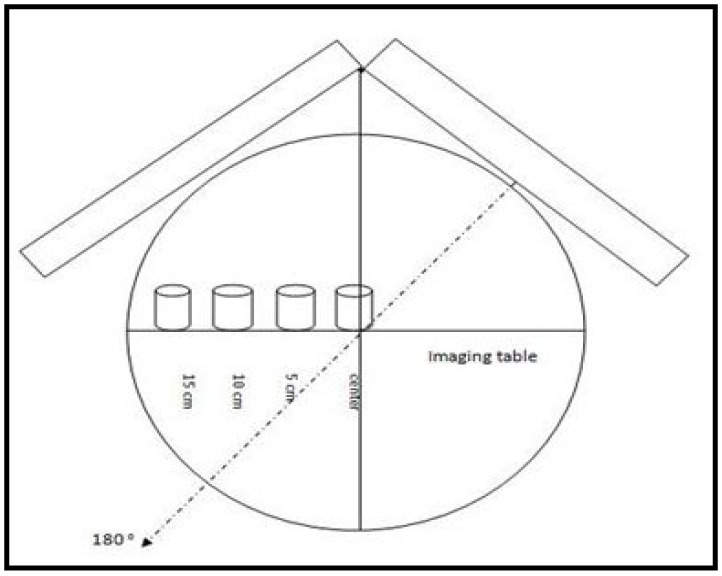
Schematic of four myocardial phantom positions on an imaging table.

The atomic number and density of the phantom material corresponded to the atomic number and density of the myocardial wall muscle (number of electrons per gram = 0.55, density = 1 g/cm^3^). The fabricated phantom was meant to simulate tissue from the myocardial wall of the left ventricle. Measurements of the fabricated phantoms of two chambers (inner and outer) were designed to mimic the left ventricle in both stages (end diastole and end systole). Non-perfused defects with a 5-mm thickness were placed in the space between the two chambers [25,26]. These defects were fixed in the mid-region of the myocardial phantom at interior and inferior locations [Figure 1]. The inner chamber of the phantom was filled with water, and the space between the two chambers was also filled with a thoroughly mixed technetium-99m solution (0.43, 0.31) (mci) (1.147 MBq) for end diastole and end systole, respectively. Moreover, a myocardial phantom was placed at four positions on an imaging table at the center and off-center (5 cm, 10 cm, and 15 cm) to mimic the normal heart position of the patient’s body [Figure 2].

### 2.2. Image Acquisition

A dual-head gamma camera model (Discovery NM/CT670 Pro) with low energy and high resolution and a parallel holes’ collimator was used in this study. At Tc-99m (140 KeV), energy windows of 15%, 20%, and 25% were used. These values were selected because they are proper energy values for clinical left ventricular measurements. An angle of 180° circular orbit from the right anterior oblique to the left posterior oblique was used. The total acquisition time was 13 min using step-shot mode. The matrix size was 64 × 64. A Butterworth filter (order 10, 0.4 frequency) was used. All acquired SPECT images of the vertical axis view were saved in DICOM format. The acquisition of SPECT images using software Image J 1.48v was quantified to filter the images during reconstruction.

For each energy window, at four positions of the phantom, six acquired SPECT slices were obtained to calculate the mean pixels. The mean pixels in the center of the defect (the coldest spot) and 10 points adjacent to the defect (5 points on each side (left–right) of the defect) were calculated. The defect contrast calculations from the short axis SPECT profile were obtained and analyzed as myocardial wall defect “hottest spot to coldest spot”. The contrast was calculated using the relation: [M-D/M], where [M] is the mean pixels’ adjacent defect and [D] is the mean pixels at the center of the defect.

Theoretically, the variability of pixels’ distribution in SPECT images should be 0%. The distribution of pixel intensity in the reconstructed SPECT images was quantitatively analyzed as the percentage as follows:

Non uniformity (%) = [(maxvalue − minvalue)/(maxvalue + minvalue)] × 100

*T*-test, *sigma plot* was used for two group comparisons between energy window image acquisition for defect contrast, which correlated with different positioning on the imaging table. Probability values of <0.05 were considered statistically significant.

The mean percentage of measurements was calculated by averaging the percentage of three repeated SPECT acquisitions-test; sigma plot was used for two groups’ comparison for end diastole and end systole image acquisitions. Probability values of <0.05 were considered statistically significant.

## 3. Results and Discussion

Myocardial perfusion imaging (MPI) using SPECT has poorer contrast for sub-endocardial defects within the myocardial wall of the left ventricle. In this study, a 5 mm thickness equals 42% of the myocardial width (12 mm) to represent the sub-endocardial defect. Results showed differences in the defect contrast value in myocardial SPECT images when different widths of the energy window (15%, 20%, and 25%) were used. Furthermore, it was found that defect contrast in anterior and inferior locations of acquired SPECT images had different values in each width of the energy window, which was used with correlation to determine the effect of the positioning on the imaging (Table 1 and Table 2).

**Figure 3:**
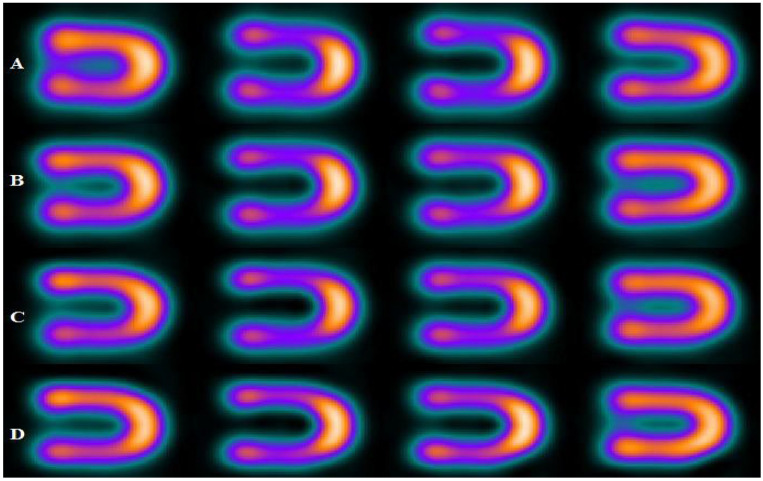
Defect in interior and inferior region of myocardial SPECT image using 15% energy window at 4 positions, where the rows (A, B, C, and D) at (the center, 5 cm, 10 cm, and 15 cm), respectively.

**Figure 4:**
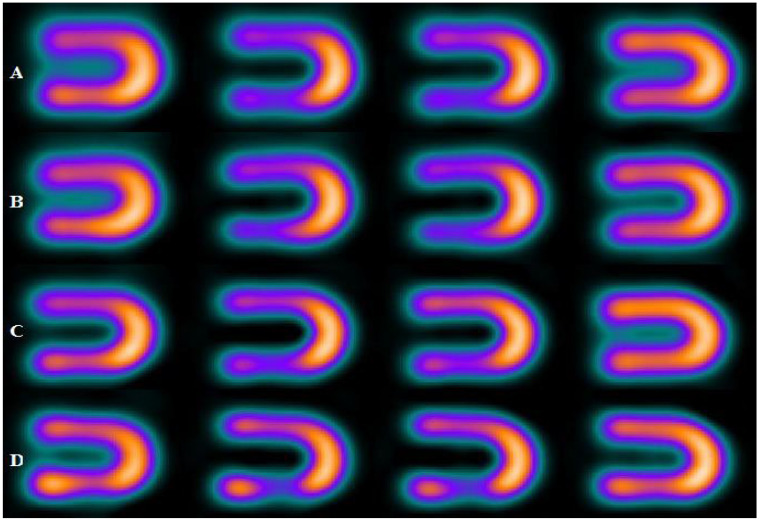
Defect in interior and inferior region of myocardial SPECT image using 20% energy window at four positions, where the rows (A, B, C, and D) at (the center, 5 cm, 10 cm, and 15 cm), respectively.

**Figure 5:**
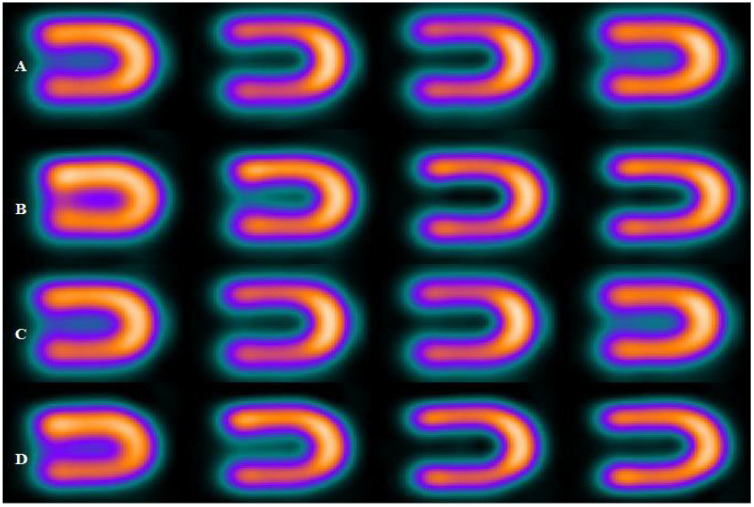
Defect in interior and inferior region of myocardial SPECT image using 25% energy window at four positions, where the rows (A, B, C, and D) at (the center, 5 cm, 10 cm, and 15 cm), respectively.

Figure 3, Figure 4 and Figure 5 represent the defect contrast using 3 energy windows at 4 positions of the phantom on an imaging table in a range of (0–15 cm) with an increment of 5 cm from the center. Visually, in the reconstruction of myocardial SPECT images, when the positioning of the myocardial phantom was at the center, the defect contrast was the greatest, whether this defect was in an anterior or inferior region within the myocardial wall phantom. Results show that the quality of the acquired images was the best when the MI phantom was positioned at the center of the imaging table, regardless of whether the defects were located in the inferior or anterior regions.

**Figure 6:**
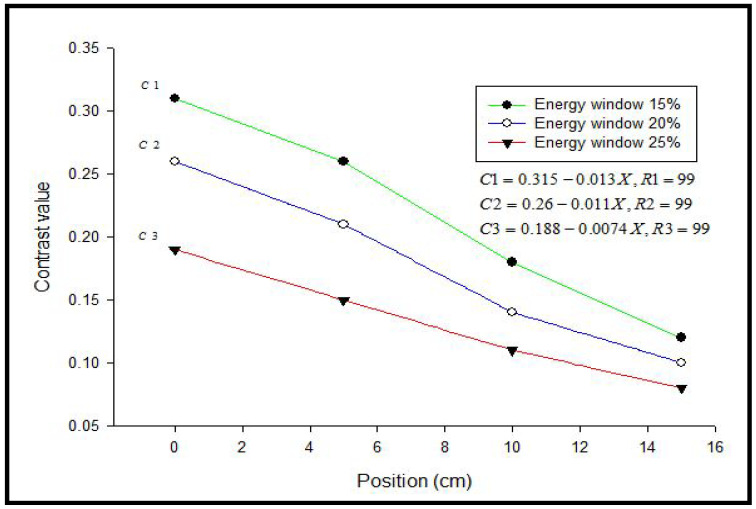
Effect of the positioning on defect contrast in the anterior region of myocardial SPECT image in different energy windows.

**Figure 7:**
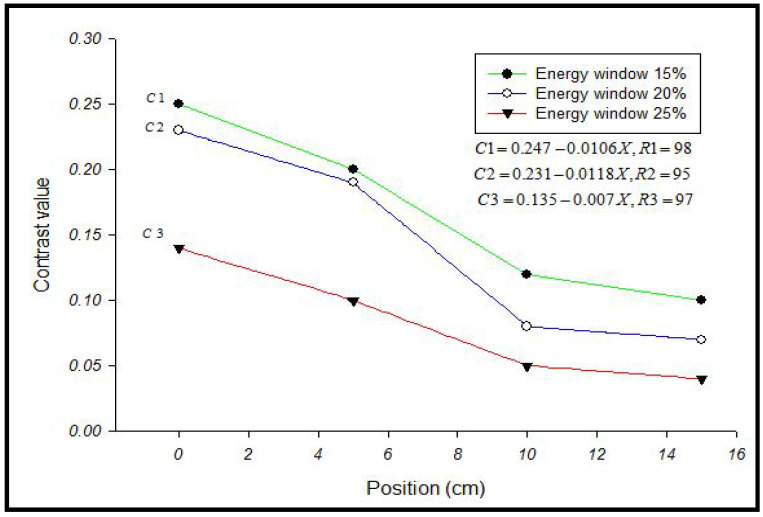
Effect of the positioning on defect contrast in the inferior region of myocardial SPECT image in different energy windows.

At the central position, results showed that the greatest defect contrast value of the 3 energy windows was (0.31) in the anterior location for a 15% energy window, while in the same window, it was (0.25) for the inferior region. Such a difference in defect contrast between anterior and inferior locations within myocardial SPECT images may be attributed to the loss of counts in the inferior region compared to the anterior [27]. When the phantom was placed off center, and the 3 energy windows were used (15%, 20%, and 25%), SPECT images showed a reduction in defect contrast in linear relation with the increase in the phantom displacement on the imaging table (R_1_, R_2_, and R_3_ = 0.99, anterior region) and (R_1_ = 0.98, R_2_ = 0.95, R_3_ = 0.97, inferior region), respectively [Figure 6 and Figure 7]. This reduction can be attributed to the decentralization of the positioning on the imaging table, which produces artifacts. This finding is in agreement with results that showed that the position of the heart within the orbit is very important and that an off-center event causes artifacts.

The reduction in defect contrast was proportional to the eccentricity of the positioning and became significant (*p* < 0.001, sigma plot) as energy windows were increased from 15% to 25% in all energy windows and positions of the myocardial phantom. The results showed a differential defect contrast between anterior and inferior locations within myocardial SPECT images, where the defect contrast was better in anterior than in inferior locations. Herein, the effects of window width (15%, 20%, and 25%) and the decentralization of the positioning on defect contrast were investigated. Moreover, the optimal defect contrast in the anterior and inferior regions of the myocardial SPECT image was improved by using energy window width with positioning. This optimal defect contrast may contribute to the improvement of SPECT protocol procedures before imaging.

If a physician could predict image distortion due to set-up error, he would be able to designate a distortion location due to blood flow insufficiency. Set-up errors cause insufficient data when the patient has been imaged sub-optimally. Therefore, another injection of a radiopharmaceutical is necessary to obtain a diagnosable image. In a clinical setting, involuntary motions (respiratory and heart motion) have a much greater impact on image quality than set-up error. In addition, SPECT image contrast is poor if the phantom is positioned off-center, even if energy-efficient windows are used.

## 4. Conclusions

The width of the wall of the MI phantom and the position on the imaging table are two important parameters in the quality assurance of myocardial SPECT imaging when the SPECT system is used to measure the thickness of MI. The results showed that the minimum detectable defect thickness in both stages (end diastole and end systole) varied in different regions in MI phantom and positions on the imaging table. Using an appropriate protocol for myocardial SPECT imaging may assess defect contrast to be at its best when an energy window of 15% is used. In conjunction with the positioning on an imaging table, the energy window width > 25% with a large displacement of phantom positioning off-center leads to loss of the defect contrast in myocardial SPECT images, particularly in an inferior region.

## Figures and Tables

**Table 1:** Values of defect contrast at the anterior location in myocardial SPECT image in different positions of the phantom for three energy windows.

Energy Window	A	B	C	D
15%	0.31	0.26	0.18	0.12
20%	0.26	0.21	0.14	0.10
25%	0.19	0.15	0.11	0.08

**Table 2:** Values of defect contrast at an inferior location in SPECT image in different positions of the phantom for three energy windows.

Energy Window	A	B	C	D
15%	0.25	0.20	0.12	0.10
20%	0.23	0.19	0.08	0.07
25%	0.14	0.10	0.05	0.05

## Data Availability

Not applicable.
