# Correction: Oglat et al. The Effect of an Energy Window with an Ellipsoid Phantom on the Differential Defect Contrast on Myocardial SPECT Images. *Bioengineering* 2022, *9*, 341

**DOI:** 10.3390/bioengineering11080844

**Published:** 2024-08-19

**Authors:** Ammar A. Oglat, Mohannad Adel Sayah, Norlaili A. Kabir

**Affiliations:** 1 Department of Medical Imaging, Faculty of Applied Medical Sciences, The Hashemite University, Zarqa 13133, Jordan; ammara@hu.edu.jo or ammar.oglat@yahoo.com; 2 Department of Radiography, Princess Aisha Bint Al-Hussein College of Nursing & Health Sciences, Al-Hussein Bin Talal University, P.O. Box 20, Ma’an 71111, Jordan; 3 School of Physics, Universiti Sains Malaysia, Penang 11800, Malaysia; norlailikabir@usm.my

Norlaili A. Kabir from the School of Physics, Universiti Sains Malaysia, Penang 11800, Malaysia was not included as an author in the original publication. [However, the main reason for the authorship change is due to unintentionally excluding Dr. Noraili A. Kabir from the list of co-authors, since she held a PhD supervising position, and she was the recipient of the financial grant which supported this research.] The corresponding author has been changed to Mohannad Adel Sayah (correspondence: 0903006@ahu.edu.jo). The corrected Author Contributions statement appears below.

**Author Contributions**: Conceptualization, A.A.O. and M.A.S.; methodology, M.A.S. and N.A.K.; software, M.A.S.; validation, A.A.O.; formal analysis, A.A.O.; investigation, M.A.S. and N.A.K.; resources, M.A.S.; data curation, A.A.O.; writing—original draft preparation, M.A.S.; writing—review and editing, A.A.O.; visualization, A.A.O.; supervision, M.A.S.; project administration, M.A.S.; funding acquisition, M.A.S. and N.A.K. All authors have read and agreed to the published version of the manuscript.

The authors state that the scientific conclusions are unaffected. This correction was approved by the Academic Editor. The original publication [1] has also been updated.
